# Interleukin-6-Mediated-Ca^2+^ Handling Abnormalities Contributes to Atrial Fibrillation in Sterile Pericarditis Rats

**DOI:** 10.3389/fimmu.2021.758157

**Published:** 2021-12-16

**Authors:** Jie Liao, Shaoshao Zhang, Shuaitao Yang, Yang Lu, Kai Lu, Yuwei Wu, Qiongfeng Wu, Ning Zhao, Qian Dong, Lei Chen, Yimei Du

**Affiliations:** ^1^ Department of Cardiology, Union Hospital, Tongji Medical College, Huazhong University of Science and Technology, Wuhan, China; ^2^ Research Center of Ion Channelopathy, Union Hospital, Tongji Medical College, Huazhong University of Science and Technology, Wuhan, China; ^3^ Institute of Cardiology, Union Hospital, Tongji Medical College, Huazhong University of Science and Technology, Wuhan, China; ^4^ Key Lab for Biological Targeted Therapy of Education Ministry and Hubei Province, Union Hospital, Tongji Medical College, Huazhong University of Science and Technology, Wuhan, China; ^5^ Department of Cardiology, Sichuan Provincial People’s Hospital, University of Electronic Science and Technology of China, Chengdu, China; ^6^ Department of Physiology, Nanjing Medical University, Nanjing, China

**Keywords:** Interleukin-6, postoperative atrial fibrillation, calcium handling abnormalities, ryanodine receptor, alternans

## Abstract

Pre-existing Ca^2+^ handling abnormalities constitute the arrhythmogenic substrate in patients developing postoperative atrial fibrillation (POAF), a common complication after cardiac surgery. Postoperative interleukin (IL)-6 levels are associated with atrial fibrosis in several animal models of POAF, contributing to atrial arrhythmias. Here, we hypothesize that IL-6-mediated-Ca^2+^ handling abnormalities contribute to atrial fibrillation (AF) in sterile pericarditis (SP) rats, an animal model of POAF. SP was induced in rats by dusting atria with sterile talcum powder. Anti-rat-IL-6 antibody (16.7 μg/kg) was administered intraperitoneally at 30 min after the recovery of anesthesia. *In vivo* electrophysiology, *ex vivo* optical mapping, western blots, and immunohistochemistry were performed to elucidate mechanisms of AF susceptibility. IL-6 neutralization ameliorated atrial inflammation and fibrosis, as well as AF susceptibility *in vivo* and the frequency of atrial ectopy and AF with a reentrant pattern in SP rats *ex vivo*. IL-6 neutralization reversed the prolongation and regional heterogeneity of Ca^2+^ transient duration, relieved alternans, reduced the incidence of discordant alternans, and prevented the reduction and regional heterogeneity of the recovery ratio of Ca^2+^ transient. In agreement, western blots showed that IL-6 neutralization reversed the reduction in the expression of ryanodine receptor 2 (RyR2) and phosphorylated phospholamban. Acute IL-6 administration to isolated rat hearts recapitulated partial Ca^2+^ handling phenotype in SP rats. In addition, intraperitoneal IL-6 administration to rats increased AF susceptibility, independent of fibrosis. Our results reveal that IL-6-mediated-Ca^2+^ handling abnormalities in SP rats, especially RyR2-dysfunction, independent of IL-6-induced-fibrosis, early contribute to the development of POAF by increasing propensity for arrhythmogenic alternans.

## Introduction

Postoperative atrial fibrillation (POAF), the most common complication of cardiac surgery, the prevalence of which varies between 20% and 40% in different studies, usually occurs 2-4 days after surgery, causing substantial increases in lengths and costs of hospital stay (1). Local inflammation, which is related to surgical lesions and postoperative pericarditis, is a major transient factor that may trigger POAF (2–6). Some clinical observations suggest that interleukin (IL)-6, a crucial pro-inflammatory cytokine, is elevated in patients with POAF (7–9). Moreover, IL-6 is early upregulated in the atria of rat and canine sterile pericarditis (SP), which are animal models of POAF (2–5). We found that IL-6 predisposes to atrial fibrosis and fibrillation by inducing cardiac fibroblast activation in SP rats (3, 4), which is further confirmed by Liu et al. (6).

Abnormalities in intracellular Ca^2+^ handling have been considered to play an important role in the initiation and maintenance of atrial fibrillation (AF) (10, 11). Recent studies reveal pre-existing altered Ca^2+^ handling, including dysfunction of ryanodine receptor 2 (RyR2) (12) or sarcoplasmic reticulum Ca^2+^-ATPase (SERCA) (13), in the atrial cardiomyocytes of POAF patients. Of note, RyR2 dysfunction can increase the risk of proarrhythmic delayed afterdepolarizations (12). Reduced SERCA weakens Ca^2+^ reuptake into the sarcoplasmic reticulum (SR), which is considered a major contributor to impaired preoperative atrial contractile function and the pre-existing arrhythmogenic substrate in patients developing POAF (13). In addition, atrial Ca^2+^ handling abnormalities, containing altered SR Ca^2+^ handling, early increase trend to Ca^2+^ transient (CaT) alternans, which may serve as a trigger for atrial arrhythmias (14). Above all, these results indicate alterations in Ca^2+^ handling may play a role in the development of POAF. A series of studies suggest that IL-6-induced negative inotropy in adult rat ventricular cardiomyocytes is mediated by the IL-6-induced inhibition of SR function (15–17). Taken together, we hypothesize that IL-6-mediated-Ca^2+^ handling abnormalities contribute to the early onset of AF in SP rats.

The hypothesis was tested in SP rats on day 3 following surgery by using anti-rat-IL-6 antibody and IL-6, with a combination of *in vivo* electrophysiology, atrial optical mapping [Ca^2+^/transmembrane potential (Vm)], and atrial fibrosis assessment. Our results suggest that IL-6-mediated-Ca^2+^ handling abnormalities in SP rats, especially RyR2 dysfunction, independent of IL-6-induced-fibrosis, early contribute to the development of POAF by increasing propensity for frequency-induced arrhythmogenic CaT alternans.

## Materials and Methods

### Preparation of the SP Model

Adult male Sprague-Dawley rats weighing 180 to 220 g were used for investigation. All experiments involving animals were approved by the Animal Research Ethics Committee of Tongji Medical College, Huazhong University of Science and Technology (IACUC Number: 2307) and were carried out in accordance with the National Institutes of Health Guide for the Care and Use of Laboratory Animals (NIH Publication, revised 2011). SP rats were created as depicted in our previous studies (2–5). Briefly, rats were anesthetized by sodium pentobarbital (i.p.; 60 mg/kg). Adequate anesthesia was assured by the absence of reflexes, and then the atria were exposed through the left second intercostal space. After a pericardiotomy, sterile talcum powder was dusted on both atrial surfaces. Sham-operated rats underwent the same procedure without pericardiotomy.

### Administration of Goat Anti-Rat-IL-6mAb, Normal Goat IgG, or IL-6

In a subset of animals, either goat anti-rat-IL-6mAb (R&D Systems, 16.7 μg/kg) or normal goat IgG (R&D Systems, 16.7 μg/kg) was administered intraperitoneally to SP rats at 30 min after the recovery of anesthesia. In another subset of animals, either recombinant rat IL-6 (PeproTech, 0.075 μg/kg/day) or the same solvent (PBS, served as controls) was administered intraperitoneally to normal rats for 3 days.

### mRNA Sequencing

Total RNAs were isolated from the atria of sham and SP rats using TRIzol reagent (Invitrogen) according to the manufacturer’s protocol. Libraries were prepared using the TruSeqTM RNA sample preparation kit (Illumina, San Diego, CA). cDNA was reverse-transcribed from mRNA using SuperScript double-stranded cDNA synthesis kit and then sequenced using the Illumina HiSeq xten/NovaSeq 6000. All sequencing data are available through the NCBI Sequence Read Archive under the accession number PRJNA747174. The quality of raw and trimmed fastq reads was checked using Hisat2. Trimming of raw data was performed using the Stringtie tool. DESeq2 was used to analyze the differentially expressed genes between samples (18). Only obvious genes with differential expression changes of > ± 1-fold change in SP versus sham samples and adjusted P value<0.05 were included in heat maps, which display raw read count values.

### *In Vivo* Electrophysiology

On the 3rd postoperative day, electrophysiology was performed as previously described (2). The atrial stimulation was performed by inserting a 6 French 10 pole coronary sinus electrode catheter into the esophagus. To test the inducibility of atrial arrhythmias, 5 consecutive bursts of rapid stimulation (25, 30, 40, 60, and 83 Hz) for 30 s at 3-minute intervals were performed through catheter electrodes. The successful AF induction was defined as a period of rapid irregular atrial rhythm for at least 1 s. The total time of AF episodes was defined as the sum of the AF duration of each episode. The probability of AF induction was determined by calculating the number of AF episodes divided by the number of total procedures. Other standard electrophysiological parameters, including threshold stimulus, Wenckebach periodicity (WP), sinus node recovery time (SNRT), rate corrected SNRT (CSNRT), and atrioventricular (AV) nodal refractory periods (AVNRPs) were also measured.

### Atrial Histology and Immunohistochemical Staining

Tissue samples from the atria were fixed with 4% paraformaldehyde, embedded in paraffin, and then sliced into 4-μm-thick sections. Some sections were stained with Masson trichrome to evaluate atrial fibrosis. A separate group of sections was immunostained with primary antibodies against rat MPO (ab208670, Abcam), CD68 (BA3638, Boster), or α-SMA (BM0002, Boster) followed by incubation with biotin-conjugated secondary antibodies, and then treated with avidin-peroxidase (2). The reaction was conducted with the DAB substrate kit (BioSci, Wuhan, China). The samples were observed at × 400, and four visual fields were analyzed in each sample using ImagePro 6.0 software.

### Western Blot Analysis

Western blots were conducted as described previously (2, 3). Total protein samples were extracted from atrial tissues and quantified using BCA protein assay kit (Servicebio, Wuhan, China). An equal amount of protein lysates was loaded to SDS-PAGE gels and then transferred onto a nitrocellulose membrane. The membranes were blocked with 5% nonfat milk and incubated with antibodies against the following proteins: IL-6 (1:1000, #12912, Cell Signaling Technology), RyR2 (1:1000, #MA3916, Thermo Fisher Scientific), p-RyR2 (Ser2808) (1:500, #A010-30AP, Badrilla), p-RyR2 (Ser2814) (1:1000, #A010-31AP, Badrilla), SERCA (1:1000, #DF6240, Affinity), PLB (1:1000, #MA3922, Thermo Fisher Scientific), p-PLB (Thr17) (1:2000, #A010-13, Badrilla), p-PLB (Ser16) (1:5000, #A010-12, Badrilla), NCX (1:1000, #55075-1-AP, Proteintech), Ca_V_1.2 (1:500, #21774-1-AP, Proteintech), IP3R (1:500, #19962-1-AP, Proteintech), and GAPDH (1:3000, ANT012s, Antegen). Corresponding secondary antibodies conjugated to horseradish peroxidase (1:5000, ANT019/ANT020, Antegen) were used for detection. Staining was detected using chemiluminescence (New Cell & Molecular Biotech, Suzhou, China) and quantified by Image Lab software (Bio-Rad, Richmond, CA, USA). GAPDH was used as a loading control.

### SERCA Activity Assay

In line with a previous research (19), SERCA activity was detected using an ultra-micro Ca^2+^-ATPase kit (Nanjing Jiancheng Bioengineering Institute, China). Total protein samples were extracted from atrial tissues. Protein concentrations were determined by using a BCA protein assay kit. SERCA activity was calculated as nmol of Pi produced per min per mg of protein.

### Optical Mapping of Ca^2+^ and Vm

On day 3 after surgery, the rats were euthanized with pentobarbital sodium containing 120 IU of heparin. Hearts were excised and Langendorff-perfused at 10-11 mL/min and 37 ± 0.5°C (20), with Tyrode solution containing (mM): 128.2 NaCl, 4.7 KCl, 1.3 CaCl_2_, 1.05 MgCl_2_, 1.19 NaH_2_PO_4_, 20 NaHCO_3_, and 11.1 glucose (pH = 7.4 adjusted with 95% O_2_/5% CO_2_). An electrocardiogram (ECG) was recorded continuously, and the pacing was performed from the right atria. Blebbistatin (10 µM), an excitation-contraction uncoupler, was added to the perfusate to eliminate motion artifacts during optical recording (21). Hearts were stained with voltage-sensitive dye [RH237, Invitrogen, Carlsbad, CA, 10 µL of 5 mg/mL in dimethylsulfoxide (DMSO)] for 5 minutes and an intracellular Ca^2+^ indicator (Rhod-2AM, Invitrogen, Carlsbad, CA, 100 µL of 1 mg/mL in DMSO and containing 20% pluronic acid) for 30 minutes. Excitation light was produced with two LED light sources at 530 ± 25 nm and bandpass filtered from 511-551 nm (LEDC-2001, MappingLab, UK). Emission light from the left atria was collected through an objective, and the signals were split with a dichroic mirror at 630 nm. The longer wavelength moiety containing the Vm signal was filtered at 700 nm, and the shorter wavelength signal moiety containing the intracellular Ca^2+^ signal was bandpass filtered with a 32 nm filter at 590 nm, as previously mentioned (22). The emitted fluorescence signals were acquired at a sampling rate of 1 kHz from a 6.4×6.4 mm field of view (64×64 pixels) with CMOS cameras (OMS-PCIE-2002, MappingLab, UK).

Baseline electrophysiological parameters were measured at a cycle length of 142.86 ms. To induce action potential duration (APD) and CaT alternans, continuous pacing at progressively faster frequencies between 125 ms and 55.56 ms was performed. To assess the restitution properties of action potential (AP) and CaT, an extra-stimulus (S1S2; 30 S1 stimuli at 142.86 ms followed by a premature S2 stimulus ranging from 140 to 35 ms) protocol was implemented. In a subset of animals, IL-6 (50 ng/ml) or PBS was added to the perfusate.

Data analysis was performed using a commercially available analysis program (OMapScope4.0, MappingLab, UK). Optical signals were spatially aligned and processed using a Gaussian spatial filter (3×3 pixels). Activation time was determined as the maximum departure velocity of Ca^2+^ upstroke. Time to peak was defined as the time from initiation of Ca^2+^ departure to the peak fluorescence, and the time to peaks from different groups were compared after a signal smoothing and spatial integration, which makes the measured value larger than the actual one (23). APD at x% repolarization (APDx) was calculated. Similarly, Ca^2+^ transient duration at x% recovery (CaDx) was calculated. Recovery of relative Ca^2+^ release in response to premature stimuli (namely RyR2 refractoriness) was measured as the ratio of the S2-induced SR Ca^2+^ release amplitude to the S1 release amplitude at S2 intervals ranging from 140 to 60 ms. Conduction velocity (CV, the speed at which the activation wavefront spreads across the atrium) was measured by the space-time coordinates of local activation. The magnitude of APD alternans was calculated as the duration difference between average longer APD80 and average shorter APD80. The spectral method was used to detect the presence of CaT alternans, which is consistent with previously described procedures (22). Accordingly, the magnitude of CaT alternans was calculated as 1 minus the ratio of the average smaller beat release amplitude to the average larger beat release amplitude.

### Statistical Analysis

Data analysis was performed using OriginPro 2018 (OriginLab Corporation, Northampton, MA, USA). Statistical significance was usually determined by a Student t-test (for comparison between sham and SP) and one-way ANOVA with Bonferroni’s *post-hoc* test (for comparison among SP, IgG, and anti-IL-6). Chi-square test was used to compare the incidence of spatially discordant alternans. Data are presented as mean ± standard error of the mean. P < 0.05 was considered statistically significant.

## Results

### IL-6 Neutralization Suppresses AF Susceptibility in SP Rats

As is shown in **Supplemental Figure 1A**, compared with sham rats, several upregulated genes in SP rats involved in inflammation, including IL-6, IL-1β, and MMP, were identified by mRNA sequencing, which is consistent with our previous findings (2–5). We further verified the increase in the protein expression levels of IL-6 in SP atria (**Supplemental Figures 1B, C**). However, the plasma IL-6 levels remained unchanged in SP rats (62.02 ± 2.33 pg/ml *vs* 70.46 ± 7.65 pg/ml, P > 0.05, n = 6/group) on day 3 after surgery.

**Figure 1:**
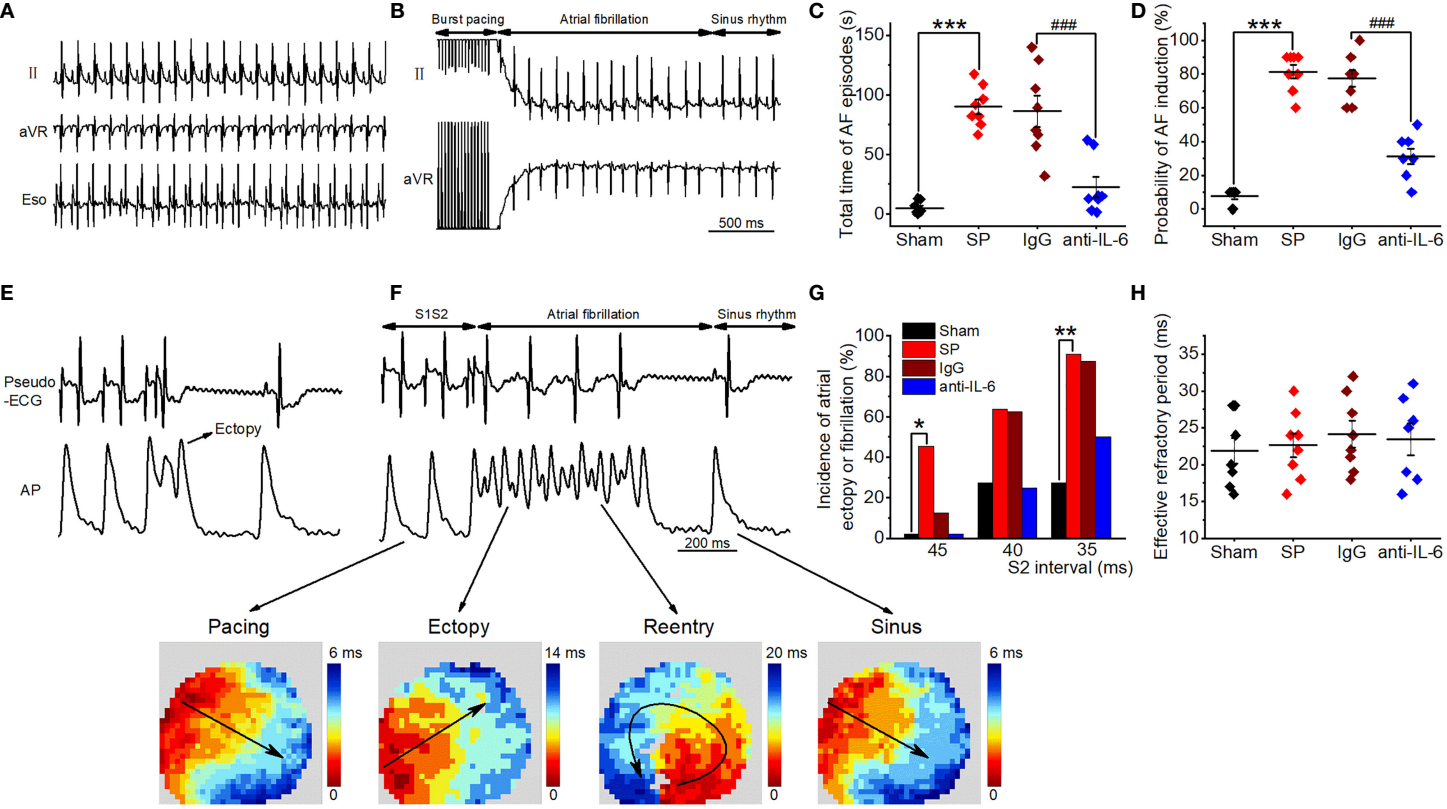
IL-6 neutralization reduces AF induction and duration in SP rats. **(A)** Representative recordings of surface and esophageal ECG during sinus rhythm. **(B)** Representative recordings of AF ECG followed by burst pacing in the SP group. **(C, D)** Statistical results of AF duration **(C)** and probability **(D)** in the Sham, SP, IgG, and anti-IL-6 groups. n = 8/group. **(E, F)** Representative AP trace and ECG recorded from an SP rat, showing atrial ectopy **(E)** and fibrillation (**F**, upper panel) induced by an extrastimulus (S1S2; S2 intervals ranging from 45 to 35 ms) method. Activation maps of pacing, ectopy, reentry, and sinus rhythm (**F**, lower panel) corresponding to the AP trace. **(G)** Incidence of atrial ectopy or fibrillation for each S2 interval. Sham, *n* = 11; SP, *n* = 11; IgG, *n* = 8; anti-IL-6, *n* = 8. **(H)** Quantification of atrial effective refractory period. Sham *n* = 9; vehicle *n* = 8; IgG, *n* = 8; anti-IL-6, *n* = 7. Statistical analyses: χ2 test for incidence, Student t-test or one-way ANOVA with Bonferroni’s *post-hoc* test for the rest. ^*^ *P* < 0.05, ^**^ *P* < 0.01, ^***^ *P* < 0.001 *vs*. Sham; ^###^ *P* < 0.001 *vs*. IgG.

We then examined the role of IL-6 in AF susceptibility by treating SP rats with an anti-rat-IL-6 antibody. Aside from the fact that the QT interval was prolonged after surgery, which may be caused by SP, there were no differences in the basic ECG parameters and the cardiac electrophysiology data measured on the 3rd postoperative day among all groups (**Supplemental Table 1**). Typical surface and esophageal ECG recordings are shown in **Figure 1A**. Surface ECG recordings from an SP rat shows induced AF after transesophageal burst pacing and spontaneous AF termination (**Figure 1B**). The total AF duration was strikingly longer (P<0.001) and the probability of induced AF was strikingly higher (P < 0.001) in SP rats than in sham rats (**Figures 1C, D**), consistent with our prior studies (2–5). Administration of anti-rat-IL-6 antibody significantly reduced the duration (P < 0.001) and probability of induced AF (P < 0.001). IgG did not improve the above parameters.

The vulnerability to atrial arrhythmia was as well validated in isolated hearts utilizing optical mapping and S1S2 stimulation. Typical optical AP traces and ECG simultaneously recorded from an SP rat are shown in **Figures 1E, F**, which demonstrate a series of arrhythmic responses, including atrial ectopy and circular reentry observed during AF by analyzing activation patterns. Representative AP traces from IgG and anti-rat-IL-6 antibody groups were shown in **Supplemental Figure 2**. Similarly, the hearts of SP rats displayed more frequent atrial ectopy or fibrillation in response to S1S2 stimulation, which was attenuated after treatment with anti-rat-IL-6 antibody (**Figure 1G**). IgG did not influence atrial arrhythmia vulnerability. There was no difference in the atrial effective refractory period (**Figure 1H**).

### IL-6 Neutralization Reduces Atrial Inflammation and Fibrosis in SP Rats

**Figure 2:**
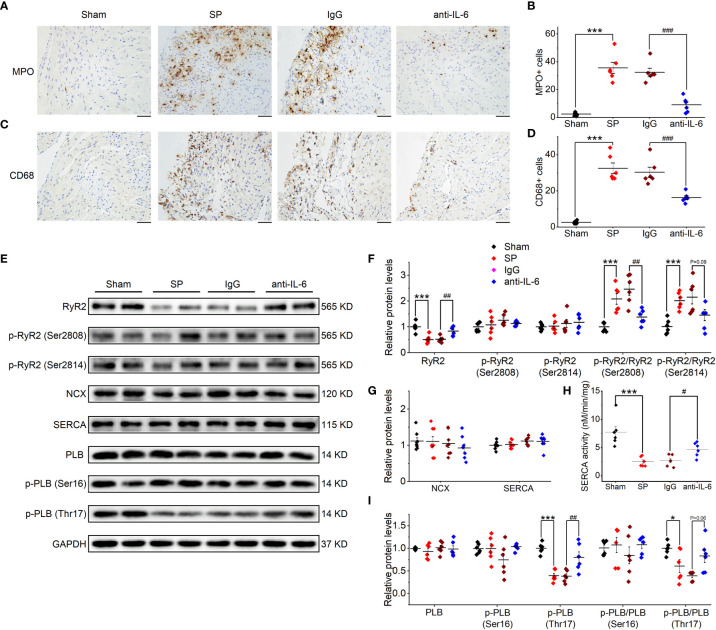
IL-6 neutralization prevents atrial inflammation and the alterations in Ca^2+^ handling proteins in SP rats. **(A, B)** Representative images of atrial MPO staining **(A)** and quantification **(B)** in the 4 indicated groups. Five visual fields are taken in each sample, and the number of immune cells from these fields is quantified with ImagePro 6.0 software and is averaged to make a statistical analysis. *n* = 6-7/group. Scale bar: 50 µm. **(C, D)** Representative images of atrial CD68 staining **(C)** and quantification **(D)**. *n* = 6-7/group. Scale bar: 50 µm. **(E–I)** Original Western blot **(E)** and quantification of the expression of RyR2, p-RyR2 (Ser2808), p-RyR2 (Ser2814) **(F)**, the expression of SERCA and NCX **(G)**, SERCA activity **(H)**, the expression of PLB, p-PLB (Ser16), and p-PLB (Thr17) **(I)**, in atrial tissue of 4 indicated groups. *n* =6-8/group. ^*^P < 0.05, ^***^P < 0.001 *vs*. Sham; ^#^P < 0.05, ^##^P < 0.01, ^###^P < 0.001 *vs*. IgG, determined by Student t-test or one-way ANOVA with Bonferroni’s *post-hoc* test. **(F–H)**.

According to several reports (2, 3, 24), SP animals are characterized by the infiltration of immune cells and fibrosis in the atria. In the current study, the infiltration of MPO+ cells (neutrophils) and CD68+ cells (macrophages) were inhibited after treatment with anti-rat-IL-6 antibody (both P < 0.001; **Figures 2A–D**). In addition, administration of anti-rat-IL-6 antibody reduced interstitial fibrosis (evaluated by Masson’s trichrome staining) and α-SMA (evaluated by immunostaining staining) in SP rats (P < 0.001, and P < 0.05, respectively; **Supplemental Figure 3**). IgG did not alter the above indices.

### IL-6 Neutralization Reverses Ca^2+^ Handling Abnormalities in SP Rats

As shown in **Supplemental Figure 4**, the atrial expression of RyR2 was reduced following SP, as suggested by mRNA sequencing. Then, we further verified the changes in atrial proteins related to Ca^2+^ handling through the use of western blot (**Figure 2E**). SP significantly reduced the expression of total RyR2, without effects on Ser2808-phosphorylated RyR2 or the Ser2814-phosphorylated form. Thus, phosphorylation ratios (Ser2808- and Ser2814-phosphorylated RyR2 to total RyR2) (**Figure 2F**) were increased in SP rats. The expression of Ca_V_1.2 was decreased in SP rats (**Supplemental Figures 5A, B**), which is consistent with our previous findings of reduced L-type calcium current in SP rats (2). Phospholamban (PLB) and the Ser16-phosphorylated form were not significantly affected by SP; nor were SERCA and Na^+^/Ca^2+^ exchanger (NCX) (**Figure 2G**). However, Thr17-phosphorylated PLB, phosphorylation ratios (Thr17-phosphorylated PLB to total PLB), and SEARA activity were decreased (**Figures 2H, I**). These alterations were reversed by treatment with the anti-rat-IL-6 antibody, but not by treatment with IgG. Interestingly, we found that the expression of IP3R was upregulated in SP rats but remained a high level after treatment with the anti-rat-IL-6 antibody (**Supplemental Figures 5A, B**).

**Figure 3:**
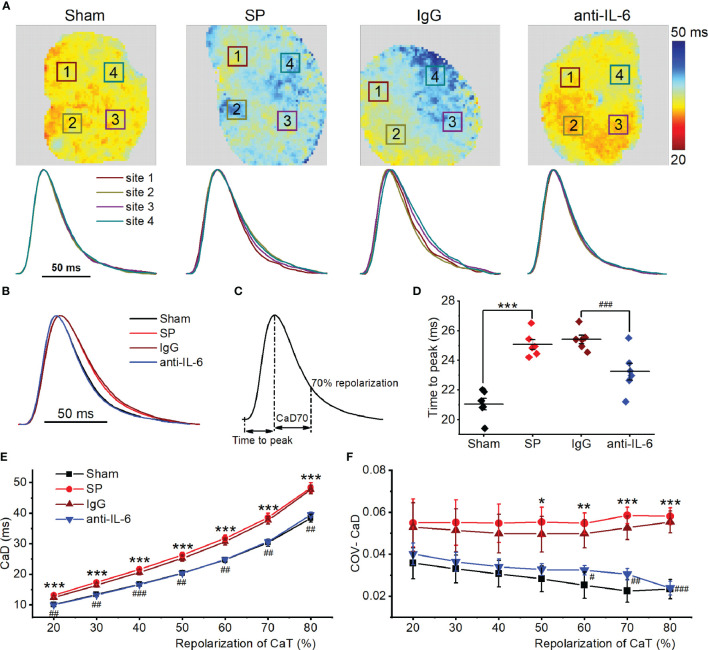
IL-6 neutralization improves atrial Ca^2+^ handling abnormalities in SP rats. **(A)** Atrial CaD70 maps from the 4 indicated groups at a cycle length of 142.86 ms (upper panel), and CaT traces from corresponding the 4 locations indicated with a box in the upper map (lower panel). **(B)** CaT traces from the 4 groups. **(C)** Calculation diagram of CaD. **(D)** Quantification of time to peak. **(E, F)** Quantification of CaD **(E)** and its coefficient of variation **(F)**. *n* = 6/group. ^*^P < 0.05, ^**^P < 0.01, ^***^P < 0.001 *vs*. Sham; ^#^P < 0.05, ^##^P < 0.01, ^###^P < 0.001 *vs*. IgG, determined by Student t-test or one-way ANOVA with Bonferroni’s *post-hoc* test.

Next, atrial SR Ca^2+^ handling was assessed in isolated hearts using optical mapping with atrial pacing at 142.86 ms. Typical CaD70 maps, along with corresponding CaT traces at 4 sites under each condition, are shown in **Figure 3A**. Time to peak and CaD in SP rats were longer than those of sham rats (**Figures 3B–E**). Spatial inhomogeneity of CaD was quantified as the coefficient of variation (COV) of CaD. COV of CaD was increased in SP rats (**Figure 3F**). Treatment with anti-rat-IL-6 antibody improved the above parameters.

### IL-6 Neutralization Normalizes Alterations in AP in SP Rats

We previously found that atrial APD in SP rats is significantly prolonged, accompanied by increased COV of APD, but the time to peak of AP is unchanged (2). All of these alterations returned to almost normal levels following treatment with anti-rat-IL-6 antibody (**Supplemental Figure 6**). Representative activation maps are exhibited in **Supplemental Figure 7A**. No difference was observed in conduction velocity at various cycle lengths (142.86 ms, 83.33 ms, and 55.56 ms) among the 4 groups (**Supplemental Figure 7B**).

### IL-6 Neutralization Alleviates Atrial Alternans in SP Rats

**Figure 4:**
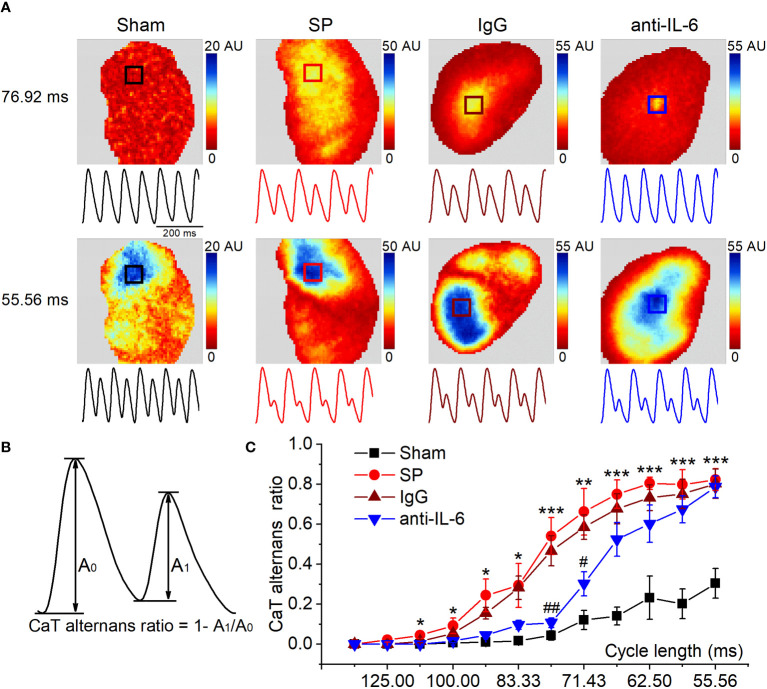
IL-6 neutralization alleviates the onset of CaT alternans in SP rats. **(A)** Maps of atrial CaT alternans (spectral magnitude) in the 4 indicated groups during progressive decreases in cycle length, along with corresponding example CaT traces (amplitude normalized to large SR Ca^2+^ release to demonstrate alternan progression) from the location indicated with a box in the maps. **(B)** Calculation diagram of CaT alternans. **(C)** Quantification of CaT alternans ratio for each cycle length. *n* = 6/group. ^*^P < 0.05, ^**^P < 0.01, ^***^P < 0.001 *vs*. Sham; ^#^P < 0.05, ^##^P < 0.01 *vs*. IgG, determined by Student t-test or one-way ANOVA with Bonferroni’s *post-hoc* test.

Atrial alternans were induced by incrementally decreasing cycle lengths (125-55.56 ms). **Figure 4A** shows the representative maps of CaT alternans magnitude and CaT traces at 76.92 ms and 55.56 ms. **Figure 4B** shows CaT traces with large and small amplitude. The CaT alternans ratio was calculated to quantify the magnitude of CaT alternans. We found that SP rats displayed a higher CaT alternans ratio between 111.11 ms and 55.56 ms compared with the sham group. However, the CaT alternans ratios were partially relieved *via* the administration of the anti-rat-IL-6 antibody, particularly at 76.92 ms and 71.43 ms (**Figure 4C**). The frequency dependence of APD alternans is shown in **Supplemental Figure 8**. APD alternans were markedly higher in SP rats than that in sham rats at pacing rates between 111.11 and 55.56 ms. Treatment with anti-rat-IL-6 antibody significantly suppressed APD alternans between 90.91 and 55.56 ms.

**Figure 5:**
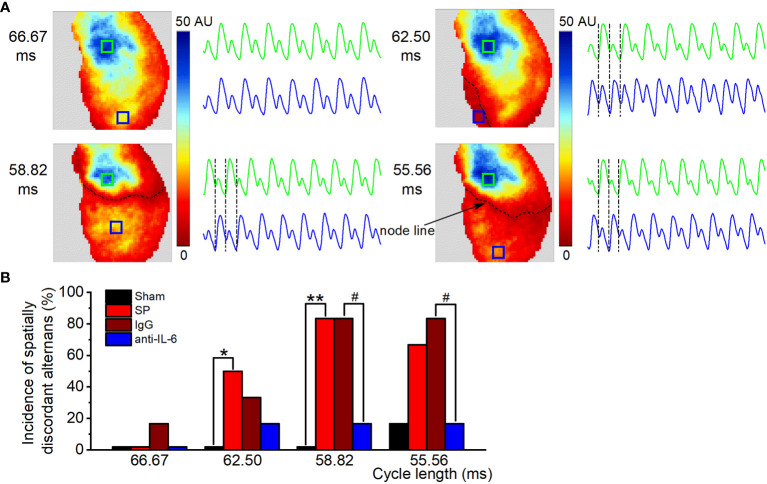
IL-6 neutralization reduces the onset of spatially discordant alternans in SP rats. **(A)** Maps of atrial CaT alternans (spectral magnitude) and example CaT traces from the corresponding locations in the maps at cycle lengths from 66.67 to 55.56 ms in the SP group, showing spatially concordant alternans at 66.67 ms and spatially discordant alternans (one area is out-phase with one another, accompanied by a node line in black between the two regions) at 62.5 ms, 58.82 ms, and 55.56 ms. **(B)** Incidence of spatially discordant alternans in the Sham, SP, IgG, and anti-IL-6 groups for each pacing rate. *n* = 6/group. ^*^P < 0.05, ^**^P < 0.01 *vs*. Sham; ^#^P < 0.05 *vs*. IgG, determined by χ2 test.

With decreases in cycle length, CaT alternans may become spatially discordant, where one area of the atrium exhibited a large-small amplitude of CaT sequence, and another area had the opposite, small-large sequence. Representative recordings of the transition from spatially concordant alternans (at 66.67 ms) to spatially discordant alternans (at 62.5-55.56 ms) from SP rats are shown in **Figure 5A**. Similar to previous findings (22, 25), we observed nodal lines between two areas with opposite phases during spatially discordant alternans. The incidence of spatially discordant alternans in SP rats was significantly higher than sham rats at 62.5-58.82 ms but was suppressed after treatment with the anti-rat-IL-6 antibody at 58.82-55.56 ms (**Figure 5B**). For example, 58.82 ms stimulation failed to induce spatially discordant alternans in the sham group but induced spatially discordant alternans in 5 of 6 SP rats. Only 2 rats developed spatially discordant alternans in the administration of the anti-rat-IL-6 antibody.

The presence of spatially discordant alternans has so far been believed to be causally associated with atrial and ventricular arrhythmia (25–27). We, therefore, investigated the relationship between zones of spatially discordant alternans and reentrant activity in SP rats (**Supplemental Figure 9**). **Supplemental Figure 9A** shows the transition from spatially concordant alternans (at 66.67 ms) to spatially discordant alternans (at 58.82 ms). **Supplemental Figure 9B** shows the pseudo-ECG, AP traces, and activation map. AF was induced in the same heart by S1S2 protocol. Please note that the location of ectopy and reentry during AF coincided closely with the site of the most obvious discordant alternans (marked with “a”), which reflected the arrhythmogenic effect of alternans.

**Figure 6:**
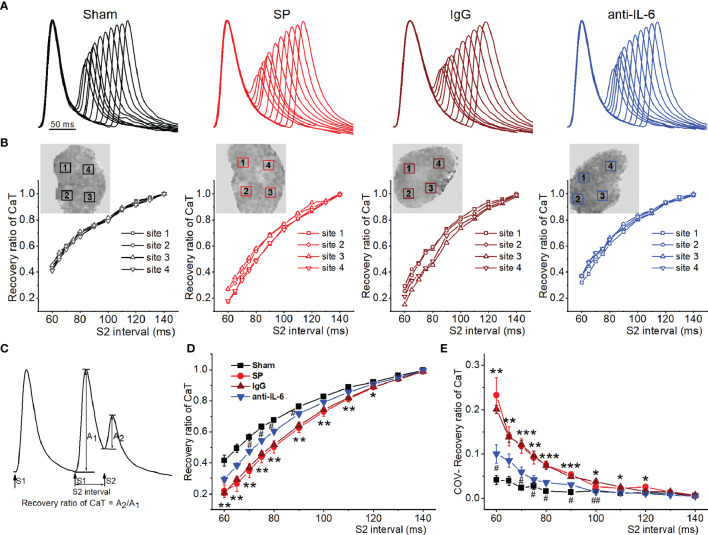
IL-6 neutralization improves atrial RyR refractoriness in SP rats. **(A)** Superimposed CaT traces from the 4 indicated groups at S2 intervals ranging from 140 to 60 ms. **(B)** The recovery ratio of CaT (A_2_/A_1_) was plotted against the S2 interval at the 4 locations indicated with a box in the upper map from the 4 groups. **(C)** Calculation diagram of recovery ratio of CaT. **(D, E)** Quantification of recovery ratio of CaT **(D)** and its COV **(E)**. *n* = 6/group. ^*^P < 0.05, ^**^P < 0.01, ^***^P < 0.001 *vs*. Sham; ^#^P < 0.05, ^##^P < 0.01 *vs*. IgG, determined by Student t-test or one-way ANOVA with Bonferroni’s *post-hoc* test.

The refractory period of CaT is commonly recognized to play a key role in the onset of CaT and APD alternans (22, 25). To quantify CaT recovery from refractoriness (A_2_/A_1_ at each S2 interval), an S1S2 protocol was used. **Figure 6A** shows representative recordings of CaT at various S1-S2 coupling intervals from each group. **Figure 6B** shows representative curves of the recovery ratio of CaT at 4 atrial locations. **Figure 6C** shows the way to calculate the recovery ratio of CaT. As shown in **Figure 6D**, the recovery ratio of CaT in SP atria displayed less than that in sham atria at S2 intervals between 60-130 ms, suggesting a delay in CaT refractoriness of the SP atria. Moreover, the COV of recovery ratio of CaT was increased in SP rats, indicating an increase in spatial inhomogeneity of CaT refractoriness (**Figure 6E**). The recovery ratio of CaT and its COV in SP rats returned to closely normal levels following treatment with anti-rat-IL-6 antibody.

### Exogenous IL-6 Administration to Isolated Rat Hearts Increases AF Susceptibility by Enhancing Vulnerability to Arrhythmogenic CaT Alternans

**Figure 7:**
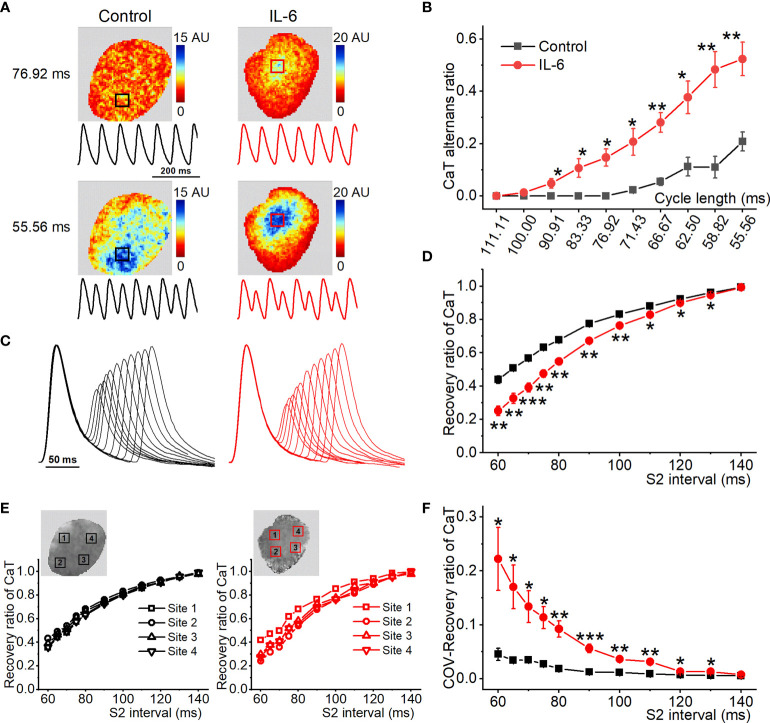
Exogenous IL-6 administration increases the onset of atrial CaT alternans. **(A)** Maps of atrial CaT alternans (spectral magnitude) in the control and IL-6 groups during progressive decreases in cycle length, along with corresponding example CaT traces from the location indicated with a box in the maps. **(B)** Quantification of CaT alternans for each pacing rate. **(C)** Superimposed CaT traces from the control and IL-6 groups at S2 intervals ranging from 140 to 60 ms. **(D)** Quantification of recovery ratio of CaT at each S2 interval. **(E)** The recovery ratio of CaT was plotted against the S2 interval at the 4 locations indicated with a box in the upper map from the 2 groups. **(F)** Quantification of COV of recovery ratio of CaT. Control, *n* = 5; IL-6, *n* = 6. ^*^P < 0.05, ^**^P < 0.01, ^***^P < 0.001 *vs*. Control determined by Student t-test.

To further reveal the causal correlation between IL-6 and CaT alternans, acute administration of exogenous IL-6 to isolated normal rat hearts was performed. **Figure 7A** shows the representative maps of CaT alternans magnitude and CaT traces at 76.92 ms and 55.56 ms. The atrium treated with PBS (used as the control) showed no CaT alternans at 76.92 ms, whereas the atrium treated with exogenous IL-6 exhibited significant CaT alternans at the same cycle length (76.92 ms). As shown in **Figure 7B**, the CaT alternans ratio was significantly increased by IL-6 at each cycle length (90.91-55.56 ms). Additionally, exogenous IL-6 administration prolonged RyR2 refractoriness, as indicated by less recovery ratio of CaT at S2 intervals between 60-130 ms (**Figures 7C, D**). Moreover, the COV of recovery ratio of CaT was markedly increased after the application of IL-6 (**Figures 7E, F**), which suggests that IL-6 increased the dispersion of RyR2 refractoriness. Although exogenous IL-6 administration slightly prolonged time to peak and CaD, they did not achieve statistical significance (**Supplemental Figures 10A–D**). However, the dispersion of CaD was strikingly increased following the administration of exogenous IL-6 (**Supplemental Figure 10E**).

**Figure 8:**
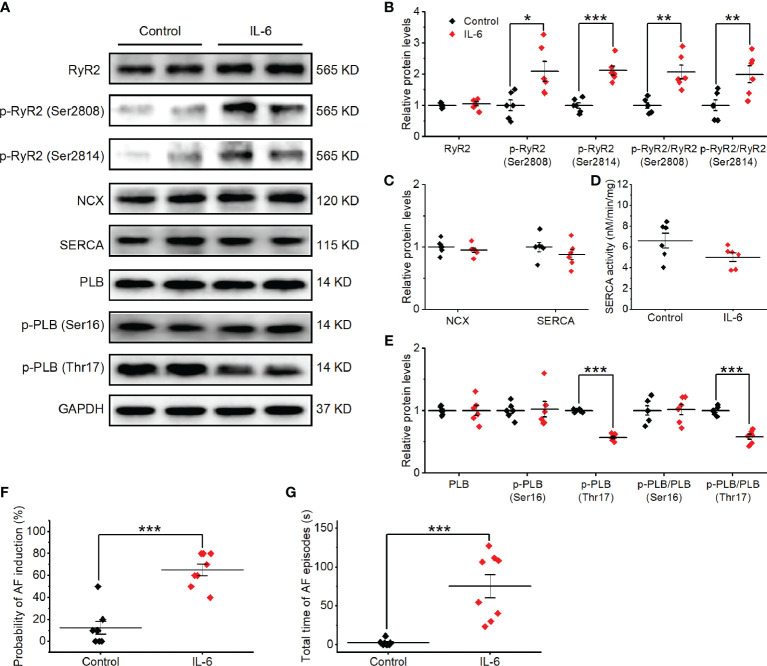
Exogenous IL-6 administration increases the alterations in Ca^2+^ handling proteins and AF induction *in vivo*. **(A–E)** Original Western blot **(A)** and quantification the expression of RyR2, p-RyR2 (Ser2808), p-RyR2 (Ser2814) **(B)**, the expression of SERCA and NCX **(C)**, SERCA activity **(D)**, the expression of PLB, p-PLB (Ser16), and p-PLB (Thr17) **(E)**, in atrial tissue of 2 indicated groups. *n* = 6/group. **(F, G)** Statistical results of AF duration **(F)** and probability **(G)** in the 2 indicated groups. *n* = 8/group. ^*^P < 0.05, ^**^P < 0.01, ^***^P < 0.001 *vs*. Control determined by Student t-test.

To gain insight into the potential molecular basis of atrial Ca^2+^ handling abnormalities caused by exogenous IL-6 administration, we performed western blot on a range of relevant proteins from atria. No changes were found in the total expression of RyR2, PLB, SERCA, or NCX. Strikingly, IL-6 administration increased the phosphorylation of RyR2 (Ser2808 and Ser2814) and reduced the phosphorylation of PLB (Thr17) and SEARA activity (**Figures 8A–E**), similar to the changes in SP rats. However, IL-6 administration had no effect on the expression of IP3R and Ca_V_1.2 (**Supplemental Figures 5C, D**).

We then sought to determine whether exogenous IL-6 administration can contribute to AF susceptibility in rats. On the 3rd day after the administration of IL-6 or PBS, no differences in the basic ECG parameters and the cardiac electrophysiology data were observed between the two groups (**Supplemental Table 2**). The total AF duration was significantly longer (P < 0.01) and the probability of induced AF was significantly higher (P < 0.01) in rats after treatment with IL-6 than treatment with PBS (**Figures 8F, G**). However, rats after treatment with IL-6 did not exhibit evident atrial fibrosis (**Supplemental Figure 11**).

## Discussion

Elevated IL-6 promotes POAF onset by increasing atrial fibrosis (3, 4, 6). However, mechanistic links between Ca^2+^ handling abnormalities related to IL-6 and POAF are still elusive. Here, the key findings are that: (i) SP rats exhibited obvious Ca^2+^ mishandling and Ca^2+^ handling heterogeneity, as well as increased susceptibility to CaT and APD alternans (particularly spatially discordant alternans) caused by a delay in the refractory period of CaT, which were causally linked to enhanced AF inducibility; (ii) IL-6 neutralization attenuated SP-induced atrial Ca^2+^ handling abnormalities and alternans; (iii) exogenous IL-6 administration elicited CaT alternans and prolonged CaT refractoriness in isolated rat hearts; and (iv) intraperitoneal IL-6 administration contributed to AF susceptibility *in vivo* without an impact on atrial fibrosis. Our results suggest that IL-6-mediated-Ca^2+^ handling abnormalities, rather than IL-6-induced-atrial fibrosis, may be a key factor in POAF pathogenesis and a potential therapeutic target for this arrhythmia.

### Ca^2+^ Handling Abnormalities and Alternans in SP Rats

When the heart is under steady-state conditions, the amount of Ca^2+^ entering the cell from SR *via* RyR2 is equivalent to the amount of Ca^2+^ pumped back up into the SR by SERCA to maintain homeostasis (28). This normal cardiac Ca^2+^ handling can be disturbed in pathological conditions (e.g., hypertension and inflammation), leading to various forms of arrhythmias, including AF (14, 29, 30). In the SP atria, the time to peak was longer, implying the dysfunction of RyR2. Dysfunctional atrial RyR2, which is predisposed to initiate atrial arrhythmias, has been reported previously in spontaneously hypertensive rats (SHR) (14) and patients with AF (31). Abnormal prolongation and dispersion of CaD suggest that the function of SR Ca^2+^ uptake was spatially impaired in SP atria, which is consistent with test results of SERCA activity. Although SR Ca^2+^ release and uptake were impaired, a new pathological balance between RyR2 release and SERCA uptake was formed in SP atria, and CaT amplitude thus varied little from beat-to-beat at 142.86 ms. Nonetheless, altered atrial Ca^2+^ handling-pathological Ca^2+^ homeostasis together with its spatial heterogeneity in SP rats constituted a substrate prone to AF.

Cardiac alternans, defined as cyclic beat-to-beat alternations in the amplitude or shape of APD and intracellular Ca^2+^ release at a constant stimulation rate, become gradually obvious with decreasing in cycle lengths (22, 32). Of note, intracellular Ca^2+^ cycling plays a major role in the development of APD alternans (i.e., CaT alternans can be a primary driver of APD alternans) (22, 25, 33). In the present experiment, APD and CaT alternans in SP atria appeared at longer cycle lengths and their severity at short cycle lengths was significantly increased compared to sham atria. Similar to SHR (14), pathological Ca^2+^ homeostasis caused by dysfunctional RyR2 and SERCA cannot adapt to fast pacing, making CaT alternans easier to occur in SP rats. As the cycle length decreased increased to 62.5-55.56 ms, CaT alternans in SP rats more easily became spatially discordant. Consistent with previous studies (25–27), heterogeneity of repolarization as spatially discordant alternans occurs in SP atria increased vulnerability to triggered and reentrant arrhythmias. Intracellular Ca^2+^ dynamics act on the ion conductances of cardiomyocytes, and thus, affect APD (32), so the heterogeneity of Ca^2+^ handling may lead to spatial differences in repolarization in our study. Previously, the mechanisms contributing to spatially discordant alternans have been identified. These include spatial heterogeneities of Ca^2+^ cycling and CV restitution (32). Therefore, our observations may be a result of spatially disturbed Ca^2+^ handling, as atrial CV did not alter in SP rats. In addition, the prolongation of APD as the result of the reduction of potassium currents, including the transient outward (*I*_to_) (2), may be associated with alternans in SP rats, but more research is required to confirm this speculation.

To gain insight into the much greater susceptibility of SP atria to alternans, we studied the refractory period of the CaT, which highly depends on the intrinsic refractoriness of RyR2 (22, 25). RyR2 refractoriness is widely regarded as a primary determinant for the onset of CaT alternans (22, 25). Strikingly, RyR2 refractoriness was delayed in the SP atria, along with evident dispersion of RyR2 refractoriness, indicating that slower recovery and regional heterogeneity of SR Ca^2+^ release may account for the occurrence of alternans (including APD-, CaT-, and spatially discordant alternans) in SP rats.

### IL-6 Neutralization Normalizes Ca^2+^ Handling Abnormalities and Alternans in SP Rats

Increased IL-6 or the activation of IL-6 signaling has been reported to contribute to the development of left ventricular diastolic dysfunction in rats or mice (34, 35), which may be partly explained by the IL-6-induced inhibition of SR function observed in adult rat ventricular cardiomyocytes (15–17). A subsequent study has shown that genetic deletion of IL-6 ameliorated cardiac dysfunction caused by pressure overload (induced by transverse aortic constriction) (34). Thus, IL-6 may be associated with Ca^2+^ mishandling in the development of diastolic dysfunction, given that cardiac systolic and diastolic function highly depend on high-efficiency Ca^2+^ handling. This study demonstrated the pathological roles of IL-6 in SP rats, linking them to Ca^2+^ handling abnormalities and alternans. Treatment with an anti-rat-IL-6 antibody not only reversed the prolongation of time to peak and CaD in SP atria but also reduced the dispersion of CaD, implying that IL-6 neutralization can normalize Ca^2+^ handling at the whole atria level by restoring dysfunctional SR Ca^2+^ release and uptake. Moreover, IL-6 neutralization attenuated APD-, CaT-, and spatially discordant alternans by reversing the delay and dispersion of RyR refractoriness.

According to a previous study, AP shortening suppresses or completely eliminates alternans in single atrial cardiomyocytes isolated from rabbits (36). In the current study, the prolongation and dispersion of APD in the SP atria, consistent with our previous finding (2), were inhibited by IL-6 neutralization. The effect of IL-6 neutralization on alterations in APD was supported by a finding that IL-6 can lengthen atrial APD in isolated rat hearts (37). Therefore, besides the recovery of Ca^2+^ handling, the effectiveness of the anti-rat-IL-6 antibody in mitigating alternans in SP atria may result from the abbreviation of APD.

### Effect of Exogenous IL-6 on Ca^2+^ Handling Abnormalities and Fibrosis

Exogenous IL-6 was acutely administrated to isolated rat hearts to test whether SP-induced Ca^2+^ handling phenotype can be reproduced in normal atria. Interestingly, exogenous IL-6 administration induced CaT alternans and prolonged CaT refractoriness, although CaD was slightly prolonged without statistical significance. However, the severity of atrial CaT alternans was inferior to that of SP, which may account for the absence of spatially discordant alternans in normal atria after IL-6 administration. We then found that intraperitoneal IL-6 administration increased AF susceptibility *in vivo*, showing a role of IL-6 in arrhythmogenesis and simulating partial SP-induced Ca^2+^ handling phenotype. Atrial fibrosis, an important mechanism for AF (38), failed to appear with IL-6 administration in our study, which was different from a previous study (35) in which IL-6 administration results in extensive cardiac fibrosis. This difference may result from low concentration (0.075 μg/kg/day *vs*. 60 μg/kg/day) and short duration (3 days *vs*. 7 days) of IL-6 administration. On all accounts, we found that altered Ca^2+^ homeostasis induced by IL-6, without atrial fibrosis, increased AF inducibility. These results suggest that the role of IL-6 in Ca^2+^ mishandling, rather than IL-6-induced-fibrosis, is a major determinant for POAF pathogenesis in SP rats.

### Molecular Basis for Ca^2+^ Handling Abnormalities

Cardiac Ca^2+^ handling is a complicated process involving a wide range of Ca^2+^ handling proteins (25, 39). In accordance with several findings (25, 40), in the current study, a reduction in total RyR2 expression underlying the dysfunction of SR Ca^2+^ release in SP rats, prolonged CaT refractoriness and promoted alternans. In addition, an interesting phenomenon that increased RyR2 Ca^2+^ leak can lead to alternans was observed in a mathematical modeling of CaT alternans (40) and canine AF model (25). Thus, upregulated RyR2 phosphorylation may enhance RyR2 Ca^2+^ leak in SP atria, aggravating alternans.

Despite no change in SERCA expression, the reduction of SERCA activity and the downregulation of PLB (Thr 17) phosphorylation might result in the dysfunction of SR Ca^2+^ uptake in the SP atria. However, the onset of CaT alternans was considered unrelated to SERCA overexpression or inhibition (41), as well as the enhancement of SERCA function by ablating PLB or the mild suppression of SERCA function (42). Thus, slowing SR Ca^2+^ uptake may not be required for CaT alternans in the SP atria. Dysfunctional NCX can also lead to slower Ca^2+^ transport out of the cell, resulting in prolonged CaD. NCX that was unchanged in the SP atria may contribute little to CaD prolongation. In the current studies, we also observed the downregulation of Ca_V_1.2 in the SP atria, which is consistent with our previous finding of reduced L-type calcium currents in atrial myocytes from SP rats (2). This alteration was reversed by treatment with anti-rat-IL-6 antibodies. However, exogenous IL-6 treatment did not affect Ca_V_1.2 expression. Treatment with anti-rat-IL-6 antibodies or IL-6 did not change the expression of IP3R. These results suggest that Ca_V_1.2 and IP3R had minimal effect on IL-6-induced Ca^2+^ handling abnormalities. The expression of NCX, Ca_V_1.2, and IP3R did not change, but their activities may alter, which deserves further study.

Strikingly, alterations in expression or modification of RyR2 and PLB in SP atria were improved with anti-rat-IL-6 antibody treatment, which is consistent with optical mapping studies. Additionally, IL-6 administration increased RyR2 phosphorylation without changing its expression, further suggesting that dysfunctional RyR2 caused by IL-6 is likely responsible for alternans.

### Limitations

Some limitations should be considered when interpreting our results. First, SP rats did not exhibit spontaneous AF, and therefore, burst atrial-pacing protocol was applied to elicit AF that lasted for a few seconds. This model might be not representative of POAF in human patients. Therefore, the beneficial effect of anti-IL-6 antibody on AF progression was confirmed in SP rats, but further human studies are needed to verify the efficacy of IL-6 neutralization. Second, it is widely recognized that, at the cellular level, Ca^2+^ handling abnormalities determined by alterations in Ca^2+^ handling proteins, resulting in proarrhythmic delayed afterdepolarizations (DADs), serve as a trigger for atrial arrhythmia. DADs may similarly occur in atrial cardiomyocytes isolated from SP rats, which need to be studied using Ca^2+^ imaging in the future. Third, how IL-6 causes changes in the proteins related to Ca^2+^ handling (e.g., RyR2 and PLB) in SP rats remains unclear. Fourth, we have only looked at the role of IL-6 in Ca^2+^ handling. It would be of interest to examine other mechanisms underlying POAF, such as atrial electrical remodeling, sympathetic activation, and other inflammatory mediators. In addition, we found that IL-6 neutralization reversed the prolongation of APD in SP rats, which may contribute to reducing AF inducibility. However, detailed information about the mechanism involved is unknown and deserves further attention.

### Conclusions

Our study provides a potential mechanistic link between IL-6 and Ca^2+^ mishandling in SP aria, which may be more important than IL-6-induced-atrial fibrosis in POAF promotion. The IL-6-mediated-Ca^2+^ handling abnormalities might constitute a novel pharmacological approach for the prevention and treatment of POAF.

## Data Availability Statement

The datasets presented in this study can be found in online repositories. The names of the repository/repositories and accession number(s) can be found below: https://www.ncbi.nlm.nih.gov/, PRJNA747174.

## Ethics Statement

The animal study was reviewed and approved by Animal Research Ethics Committee of Tongji Medical College, Huazhong University of Science and Technology.

## Author Contributions

YD and JL initially conceived the project. JL performed surgery and *ex vivo* optical mapping. SZ, SY, YL, KL, and YW carried out *in vivo* electrophysiology and western blots experiments. JL, QW, NZ, and QD analyzed the data. JL drafted the manuscript. YD and LC reviewed of the data and provided critical advice throughout the research. YD supervised and provided funding for the project. All authors revised the manuscript.

## Funding

This work was supported by grants from the National Nature Science Foundation of China to (No.81770328, No. 82170326 to YD, No. 81971274 to LC, No. 81700332 to NZ, and No. 81900324 to QW).

## Conflict of Interest

The authors declare that the research was conducted in the absence of any commercial or financial relationships that could be construed as a potential conflict of interest.

## Publisher’s Note

All claims expressed in this article are solely those of the authors and do not necessarily represent those of their affiliated organizations, or those of the publisher, the editors and the reviewers. Any product that may be evaluated in this article, or claim that may be made by its manufacturer, is not guaranteed or endorsed by the publisher.
