# Supplementary material for: Interleukin-6-Mediated-Ca2+ Handling Abnormalities Contributes to Atrial Fibrillation in Sterile Pericarditis Rats
Source: Front Immunol. 2021 Dec 16;12:758157. doi: 10.3389/fimmu.2021.758157 (PMC8716408; doi:10.3389/fimmu.2021.758157)
Supplement: Supplementary file 1 [file DataSheet_1.docx]

Interleukin-6-mediated-Ca^2+^ handling abnormalities contributes to atrial fibrillation in sterile pericarditis rats

Jie Liao ^1,2,3,4,5,#^, Shaoshao Zhang ^1,2,3,4,#^, Shuaitao Yang ^1,2,3,4,#^, Yang Lu ^1,2,3,4^, Kai Lu ^1,2,3,4^, Yuwei Wu ^1,2,3,4^, Qiongfeng Wu ^1,2,3,4^, Ning Zhao ^1,2,3,4^ , Qian Dong ^1,2,3,4^, Lei Chen^6*^, Yimei Du ^1,2,3,4*^

^1^Department of Cardiology, ^2^Research Center of Ion Channelopathy, ^3^Institute of cardiology, ^4^Key lab for biological targeted therapy of Education Ministry and Hubei Province, Union Hospital, Tongji Medical College, Huazhong University of Science and Technology, Wuhan, Hubei, China; ^5^Department of Cardiology, Sichuan Provincial People's Hospital, University of Electronic Science and Technology of China, Chengdu, China; ^6^Department of Physiology, Nanjing Medical University, Nanjing, China;

^#^ Drs. Liao, Zhang and Yang contributed equally to this work.

**^*^Corresponding authors:**

Dr. Lei Chen, E-mail: chenl@njmu.edu.cn;

Dr. Yimei Du, E-mail: yimeidu@mail.hust.edu.cn, ORCID: 0000-0003-1125-0294

**

Supplemental Figure S1** Increased expression of IL-6 in the atria of SP rats. (A) Heat map showing expression of genes related to inflammation in the 2 indicated groups. n = 3/group. Red represented a higher RPKM; blue represented a lower RPKM. Results were based on RNA sequencing samples. (B and C) Representative Western blot (B) and quantification (C) of IL-6 in atrial tissue of Sham and SP rats. n = 9/group. ^**^P < 0.01 vs. Sham determined by Student t-test.

|  | Sham | SP | IgG | anti-IL-6 |
| --- | --- | --- | --- | --- |
| HR, times/min | 464.50 ± 33.45 | 448.63 ± 27.27 | 455.25 ± 22.83 | 450.50 ± 29.37 |
| P, ms | 20.63 ± 1.03 | 19.63 ± 0.94 | 19.63 ± 0.97 | 18.94 ± 1.08 |
| PR, ms | 46.38 ± 3.36 | 46.78 ± 3.27 | 46.13 ± 2.47 | 45.56 ± 3.04 |
| QRS, ms | 14.15 ± 1.09 | 14.63 ± 0.83 | 14.35 ± 0.75 | 14.58 ± 0.96 |
| QT, ms | 65.63 ± 3.21 | 75.80 ± 2.69^***^ | 75.93 ± 7.50^***^ | 75.49 ± 7.51^***^ |
| WCL, ms | 79.70 ± 4.02 | 83.46 ± 3.86 | 83.63 ± 3.63 | 84.19 ± 3.72 |
| CSNRT, ms | 31.38 ± 2.67 | 30.88 ± 4.42 | 34.25 ± 3.62 | 33.38 ± 2.92 |
| AVNERP120, ms | 67.71 ± 3.35 | 66.13 ± 3.40 | 73.13 ± 4.05 | 69.88 ± 4.36 |
| AVNERP110, ms | 68.13 ± 3.27 | 67.00 ± 3.66 | 74.00 ± 4.07 | 72.00 ± 5.48 |
| AVNERP100, ms | 68.75 ± 2.82 | 67.63 ± 4.07 | 74.00 ± 3.93 | 71.38 ± 3.78 |
| Threshold, mA | 1.18 ± 0.13 | 1.11 ± 0.14 | 1.34 ± 0.21 | 1.33 ± 0.28 |

**Supplemental Table 1** Evaluation of surface ECG parameters, transesophageal recording, and atrial stimulation. n = 8/group. Statistical analyses: Student t-test or one-way ANOVA with Bonferroni’s post-hoc test. Data are the means ± SEM. ***P<0.001 vs. Sham. Sham, sham-operated rats; SP, sterile pericarditis rats; IgG, SP rats treated with IgG; anti-IL-6, SP rats treated with anti-rat-IL-6 antibody; ECG, electrocardiogram; HR, heart rate; WCL, Wenckebach cycle length, CSNRT, corrected sinus node recovery time; SACT, sinoatrial conduction time; AVERP, atrioventricular (AV) nodal refractory period.

**

** Supplemental Figure S2 Representative pseudo- ECG and AP trace recorded from the group of sham (A), SP (B), IgG(C), and anti-IL-6 (D).

**

**

**Supplemental Figure S3** IL-6 neutralization alleviates atrial fibrosis in SP rats. (A and B) Representative histological sections stained with Masson trichrome (A) and percentage of left atrial interstitial fibrosis (B) in the 4 indicated groups. n = 6/group. Scale bars: 50 μm. (C and D) Examples of α-SMA immunohistochemical staining (C) and quantification (D). n = 6-7/group. Scale bars: 50 μm. ^***^P < 0.001 vs. Sham; ^#^P < 0.05, ^###^P < 0.01 vs. IgG, determined by Student t-test or one-way ANOVA with Bonferroni’s post-hoc test.

**

**

**Supplemental Figure S****4** Alterations in mRNA expression related to Ca^2+^ handling in SP atria. Heat map showing expression of genes related to Ca^2+^ handling in the 2 indicated groups. n = 3/group. Red represented a higher RPKM; blue represented a lower RPKM. Results were based on RNA sequencing samples.

**

**

**Supplemental Figure S5 Effects of IL-6 on the expression of IP3R and Ca_V_1.2 in rat atria.** Original Western blot (A) and quantification(B) in atrial tissue of 4 indicated groups. n =6/group. ^**^P < 0.01, ^***^P < 0.001 vs. Sham; ^##^P < 0.01 vs. IgG, determined by Student t-test or one-way ANOVA with Bonferroni’s post-hoc test. Original Western blot (A) and quantification(B) in atrial tissue of 4 indicated groups. n =4/group, determined by Student t-test.

**

**

**Supplemental Figure S6** IL-6 neutralization normalizes alterations in AP in SP rats. (A) Atrial APD70 maps from the 4 indicated groups at a cycle length of 142.86 ms (upper panel), and AP traces from corresponding the 4 locations indicated with a box in the upper map (lower panel). (B) AP traces from the 4 groups. (C) Calculation diagram of APD. (D) Quantification of time to peak. (E and F) Quantification of APD (E) and its coefficient of variation (F). n = 6-8/group. ^*^P < 0.05, ^**^P < 0.01, ^***^P < 0.001 vs. Sham; ^#^P < 0.05, ^##^P < 0.01, ^###^P < 0.001 vs. IgG, determined by Student t-test or one-way ANOVA with Bonferroni’s post-hoc test.

**

**

**Supplemental Figure S7** IL-6 neutralization does not alter atrial conduction velocity in SP rats. (A and B) Representative atrial activation maps at a cycle length of 142.86 ms (A) and quantification of conduction velocity for each cycle length (B) in the 4 indicated groups. n = 6-8/group.

**

**

**Supplemental Figure S8** IL-6 neutralization alleviates the onset of APD-alternans in SP rats. (A) Typical AP traces (amplitude normalized to large AP amplitude to demonstrate alternan progression) from the Sham, SP, IgG, and anti-IL-6 groups during progressive decreases in cycle length. B: Calculation diagram of APD-ALT. C: Quantification of APD-ALT for each cycle length. n = 6-8/group. ^*^P < 0.05, ^**^P < 0.01, ^***^P < 0.001 vs. Sham; ^#^P < 0.05, ^##^P < 0.01, ^###^P < 0.001 vs. IgG, determined by Student t-test or one-way ANOVA with Bonferroni’s post-hoc test.

**
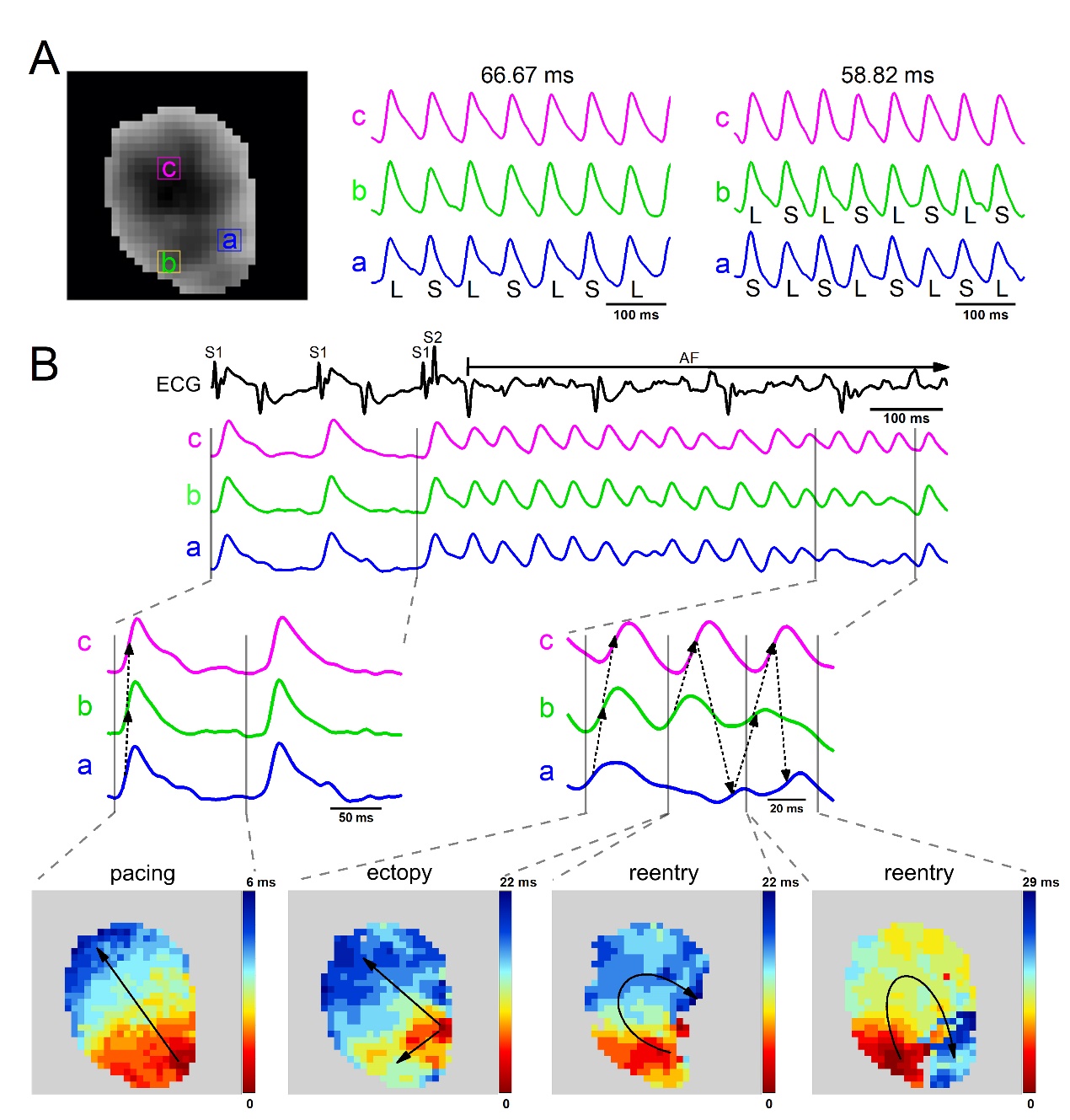
**

**Supplemental Figure S9** Trigger or reentry activity starts from the location with the most serious alternans. (A) AP traces at a cycle length of 66.67 ms and 58.82 ms, corresponding to the locations (a, b, and c) indicated with a box in the left atrial map recorded from an SP rat. APD-alternans was most obvious in location a. (B) Representative AP trace and ECG showing AF, induced by an extrastimulus method. Activation maps of ectopy and reentry corresponding to the AP traces started from location a (lower panel).

**

**

**Supplemental Figure S10** Effect of IL-6 on atrial Ca^2+^ handling. (A) Atrial CaD70 maps from the 2 indicated groups at a cycle length of 142.86 ms (upper panel), and CaT traces from corresponding the 2 locations indicated with a box in the upper map (lower panel). (B) CaT traces from the 2 groups. (C) Quantification of time to peak. (D and E) Quantification of CaD (D) and its coefficient of variation (E). Control, n = 5; IL-6, n = 6. ^*^P < 0.05, ^**^P < 0.01, ^***^P < 0.001 vs. Control determined by Student t-test.

**

**

**Supplemental Figure S11** Effect of IL-6 on atrial fibrosis. (A and B) Representative histological sections stained with Masson trichrome (A) and percentage of left atrial interstitial fibrosis (B) in the 2 indicated groups. n = 3/group. Scale bars: 50 μm. (C and D) Examples of α-SMA immunohistochemical staining (C) and quantification (D). n = 3/group. Scale bars: 50 μm.

|  | Control | IL-6 |
| --- | --- | --- |
| HR, times/min | 484.25 ± 53.95 | 463.75 ± 31.12 |
| P, ms | 19.15 ± 1.98 | 18.76 ± 1.74 |
| PR, ms | 46.24 ± 4.28 | 44.76 ± 2.58 |
| QRS, ms | 13.93 ± 0.93 | 14.59 ± 1.06 |
| QT, ms | 63.22 ± 7.69 | 61.33 ± 3.53 |
| WCL, ms | 82.45 ± 6.36 | 81.81 ± 4.67 |
| CSNRT, ms | 26.13 ± 6.13 | 27.13 ± 7.61 |
| AVNERP120, ms | 69.33 ± 6.38 | 67.33 ± 3.78 |
| AVNERP110, ms | 71.29 ± 6.02 | 67.00 ± 3.78 |
| AVNERP100, ms | 69.50 ± 4.24 | 67.50 ± 3.55 |
| Treshold, mA | 1.36 ± 0.19 | 1.2 ± 0.28 |

**Supplemental Table 2** Evaluation of surface ECG parameters, transesophageal recording, and atrial stimulation. n = 8/group. Statistical analyses: Student t-test. Data are the means ± SEM. Control, rats treated with PBS; IL-6, rats treated with IL-6.
